# Supplementary material for: The bio-sonographic index. A novel modality for early detection of acute kidney injury after complex vascular surgery. A protocol for an exploratory prospective study
Source: PLoS One. 2020 Nov 17;15(11):e0241782. doi: 10.1371/journal.pone.0241782 (PMC7671487; doi:10.1371/journal.pone.0241782)
Supplement: Attachment — Submitted filename: PONE_Rebuttal letter_1.docx [file pone.0241782.s001.docx]

Dr. Richard Hodge
Associate Editor
PLOS ONE

August 2, 2020

Re: PONE-D-20-08584
The Bio-sonographic Index. A Novel Modality for Early Detection of Acute Kidney Injury after Complex Vascular Surgery. A Protocol for an Exploratory Prospective Study
PLOS ONE

Dear Dr. Hodge,

We would like to thank the reviewers for their insightful comments on our manuscript.

We have made substantial revisions of the manuscript to address all of the reviewers’ comments. We have added 13 new references, 3 new tables, and a new figure.

Please see attached are:

1. Revised manuscript in track changes
2. Revised manuscript not in track changes
3. Rebuttal letter addressing reviewers’ comments one by one

We hope that you now find our manuscript now suitable for publication at PLOS one.

Sincerely,

Ahmed Zaky

Reviewers' comments:

Reviewer's Responses to Questions

**Comments to the Author**

1. Does the manuscript provide a valid rationale for the proposed study, with clearly identified and justified research questions?

The research question outlined is expected to address a valid academic problem or topic and contribute to the base of knowledge in the field.

Reviewer #1: Partly

Reviewer #2: Yes

Reviewer #3: Yes

2. Is the protocol technically sound and planned in a manner that will lead to a meaningful outcome and allow testing the stated hypotheses?

The manuscript should describe the methods in sufficient detail to prevent undisclosed flexibility in the experimental procedure or analysis pipeline, including sufficient outcome-neutral conditions (e.g. necessary controls, absence of floor or ceiling effects) to test the proposed hypotheses and a statistical power analysis where applicable. As there may be aspects of the methodology and analysis which can only be refined once the work is undertaken, authors should outline potential assumptions and explicitly describe what aspects of the proposed analyses, if any, are exploratory.

Reviewer #1: Partly

Reviewer #2: Yes

Reviewer #3: Yes

3. Is the methodology feasible and described in sufficient detail to allow the work to be replicable?

Reviewer #1: Yes

Reviewer #2: Yes

Reviewer #3: Yes

4. Have the authors described where all data underlying the findings will be made available when the study is complete?

The [PLOS Data policy](https://journals.plos.org/plosone/s/materials-and-software-sharing) requires authors to make all data underlying the findings described in their manuscript fully available without restriction, with rare exception, at the time of publication. The data should be provided as part of the manuscript or its supporting information, or deposited to a public repository. For example, in addition to summary statistics, the data points behind means, medians and variance measures should be available. If there are restrictions on publicly sharing data—e.g. participant privacy or use of data from a third party—those must be specified.

Reviewer #1: Yes

Reviewer #2: No

Reviewer #3: Yes

5. Is the manuscript presented in an intelligible fashion and written in standard English?

*PLOS ONE* does not copyedit accepted manuscripts, so the language in submitted articles must be clear, correct, and unambiguous. Any typographical or grammatical errors should be corrected at revision, so please note any specific errors here.

Reviewer #1: Yes

Reviewer #2: Yes

Reviewer #3: Yes

6. Review Comments to the Author

Please use the space provided to explain your answers to the questions above and, if applicable, provide comments about issues authors must address before this protocol can be accepted for publication. You may also include additional comments for the author, including concerns about research or publication ethics.

You may also provide optional suggestions and comments to authors that they might find helpful in planning their study.


(Please upload your review as an attachment if it exceeds 20,000 characters)

Reviewer #1: The authors attempt to establish a modality for early detection of acute kidney injury after complex vascular surgery and exhibit the protocol in this manuscript. I have following major comments:

1. Based on the observational nature of the study, the authors described in the section of perioperative procedures ”conventional anesthetic care, fluid, pharmacologic and blood resuscitation practices will be left to the discretion of the staff anesthesiologist and surgeon”. Multiple factors might be associated with pAKI, therefore the authors would provide the details of the perioperative procedures so that to confirm the methods difference would not affect the primary endpoint. Furthermore, the difference of the perioperative procedures might influence the sample size estimation. In a word, the authors would provide the protocol of the perioperative procedures adopted in the manuscript.

***Authors’ response: we thank the reviewer for his comment. We have addressed our anesthesia and organ protection protocols in Tables 2-4***

2. Authors used traditional marker “creatinine” as the “positive control” to help evaluating the new biomarker “IGFBP-7/TIMP-2”. Given the nature that “creatinine” was the marker of renal function, adding new biomarker related with renal injury, such as NGAL/Kim1 would be better.

***Authors’ response***

***We have added NGAL to the BSI as suggested by the reviewer.***

Reviewer #2: Dr. Zaky and colleagues outline a proposal to evaluate a novel modality for early detection of AKI. I think this is a great idea that addresses a clear need, surgery-associated AKI prediction and early detection. However, I found some of the details a bit confusing. My intent here is to ask clarifying questions to improve the manuscript, but I like the overall concept of the study.

1. At various points in the manuscript, the goal seems to vacillate from prediction and early detection of AKI to redefinition of AKI entirely. While that is a worthy long-term goal given the limitations of the current KDIGO-based definition, it is outside the scope of this project. The abstract says “an approach that is based on structural characterization of AKI is more specific and sensitive to its occurrence” for instance. I mention it here in the abstract, but it occurs at other points, and I would make sure the focus of prediction and early detection is consistent throughout.

***Authors’ response***

***We thank the reviewer for his comment. We have made substantial changes in the wording to consistently reflect the reviewer’s suggestion (Abstract, P:1, L: 28-30, P:3, L:6-7; P:13, L:19-23)***

2. I don’t think the rationale behind obtaining three different shear wave elastography measurements within hours of each other is clear. I looked at the references provided (10-13), and they all measure shear wave elastography in the setting of static processes such as fibrosis and tumor density, and not for dynamic processes such as inflammation, edema, vascular changes, necrosis, apoptosis, or other acute sorts of changes. Why do you expect to see changes in the your elastography findings, given that fibrosis and other major structural remodeling don’t happen within the timeframe proposed? It seems to me that the biggest benefit of using elastography prior to surgery is that you may identify patients with underlying fibrosis, suggestive of early “CKD” that has not yet reached an eGFR < 60 (as noted in the Introduction). I don’t see the value of the post-surgical SWE, however.

***Authors’ response***

***We thank the reviewer for his comment. Whereas, SWE has been used to detect fibrosis in a static setting, there are other factors that may lead to cortical stiffness that are not related to fibrosis and that may occur in an acute dynamic setting, such as an increase in vascularity resulting from inflammation, an increase in back pressure from the heart due to perioperative worsening of diastolic function. furthermore, we will be exploring non-stiffness renal U/S that could be of diagnostic value in an acute dynamic setting. By having each patient as his/her control we plan to identify changes in renal stiffness after the procedure compared to before procedure. Since this is the first study to assess renal stiffness in AKI, our current hypothesis is exploratory in nature. We have substantiated this concept in the discussion (P: 18, L:9-18), added 2 references (ref., 29 and 30)***

a. Minor point: I saw that the authors in their limitations section note they will assess stiffness not due to fibrosis, and cited (10). That was a bit misleading, because that study is still looking at tumor stiffness compared to normal tissue at a single point in time, rather than any sort of dynamic process.
b. Minor point: in the exploratory analyses, it notes exploring relationship between CCAs and fibrosis, which doesn’t make much physiologic sense to me. As far as I know, these CCAs don’t have much utility in CKD detection, and I’m not sure why they would based on the mechanism. The section continues talking about renal cotical tissue fibrosis changes at 3 different time points. There’s no pathophysiological basis that I can see there, either, since kidney fibrosis does not change significantly over a 6 hour period, or in the immediate post-op period as proposed. Even though these are exploratory aims, the rationale behind them needs to be clear.

***Authors’ response***

***We thank the reviewer for his comments. As indicated, SWE measures tissue stiffness of which fibrosis is only a cause. There are other causes for renal stiffness that are not due to fibrosis such as right heart diastolic dysfunction, increased tissue vascularity or an increase inflammatory response, all are factors that we would expect to observe on the SWE exam as a dynamic change from pre to postoperatively. As well, we will be observing non-stiffness-related indicators of renal inflammation in terms of vascularity and parenchyma that may all provide an additional diagnostic value in an acute setting. Exploring the relationship between renal cortical stiffness and CCA aims at exploring a relationship between radiological and biochemical evidence of inflammation at the renal cortex. As such, this is an exploratory analysis that may show a correlation. We have substantiated this concept in the discussion (P: 18, L:9-18), added 2 references (ref., 29 and 30)***

c. Minor point: You say in the limitations section that you will avoid CKD, but you only exclude GFR < 15 in the exclusions section, so are CKD 3 and 4 eligible, or not?

***Authors’ response***

***We thank the reviewer for his comments. we will include patients with eGFR > 15 ml/min/m^2^ including CKG stages 3 and 4. Only CKD stage 5 will be excluded.***

d. As for post-surgical studies, have you considered other experimental US techniques, such as those measuring cortex microvascular flow? You have a number of exploratory aims, so just a thought.

***Authors’ response***

***we thank the reviewer for his comments. We have considered the use of contrast enhanced renal U/S to assess microcirculation, yet the process of getting approval for a human study is long since it is not FDA approved for this indication as of yet. Furthermore, the technique is not feasible clinically as it is time consuming, exposes patients to contrast risks, is expensive and requires a high level of expertise that may interfere with flow dynamics of the study.***

3. The purpose of the control group is unclear to me. Standard values (4.7 kPa) exist already, and are the basis for your BSI. There is a line about evaluating for potential confounders of SWE values, although I don’t know that the controls will actually be useful in that regard. Also, are the controls getting 3 sets of US and CCA markers drawn, like in the surgery group?

***Authors’ response***

***Each patient will serve as his own control. We have changed the name of the control group into the feasibility group which will be studied to determine feasibility of performing and refining the SWE protocol and establishing reference values for normality in a sample of our population. We have also changed cutoff from an absolute value to a % change from baseline, (P:10, L:17-20)***

4. Table 1 says 90% for AKI in patients with positive tests, while the power calculations use 70%.

***Authors’ response***

Power calculation is assuming lower % of AKI (70%) in patients we should be able to detect for a given sample size. If we have 90% for AKI, we will have higher power.


5. How the two parts of the BSI work together as part of a single score is unclear to me. You have a cutoff for SWE and a cut-off for CCAs. Is BSI binary, such that having one positive (SWE or CCA) make the whole BSI positive? Is there a score that is generated?

***Authors’ response***

***We have added NGAL to the BSI and given the lack of consensus on NGAL abnormal values, have unified the BSI as a % change from baseline. We have adjusted statistics accordingly (P:10, l:2-20)***

6. For point 3 in the discussion section, I’m not sure how you are overcoming the limitations of creatinine, since you are still using creatinine to define AKI, rather than advancing a new definition of AKI (see point 1 above).
a. Sub-point: I don’t see how this metric will overcome under-representation of women and elderly in longitudinal studies, which the manuscript seems to be imply the BSI will do. I would remove the part about direct measurement of GFR, which doesn’t seem to have much to do with this study.

***Authors’ response***

***We thank the reviewer for his comment. We have removed that section form the discussion as suggested, have adjusted the discussion to align with the flow of the manuscript (Abstract, P:1, L: 28-30, P:3, L:6-7; P:13, L:19-23)***

7. I don’t understand the fourth discussion comment about proteinuria. Are you referring to how you are adjusting the CCA urine values for albumin and creatinine? That may not have been done in prior studies, but it doesn’t seem to be a unique feature of the BSI. You could adjust for albumin without using SWE.

***Authors’ response***

***We agree with the reviewer and have better explained the benefits of adjusting for urine albumin when reporting CCA values and interpretations (P:14, L:11-13).***

Reviewer #3: The study aims to use novel index (ultrasonography and biomarkers) to early detect and predict the occurrence of AKI in patients undergoing complex vascular surgery.

It is a good study to explore and if successful will have implication on the diagnosis method.

However, the manuscript requires further improvement.


Comments

Sample size calculation
Page 4 Paragraph 1, what abnormal patient and normal patient group refers to be clearly stated.

***Authors’ response***

***We apologize for the confusion. We have explained the statement more clearly (P 4, L5-11)***

Page 5 Table 1, symbol <= to be replaced with symbol ≤

Procedures
Page 5, Kpa to be standardized kPa.

It would be good to have a chart to indicate/summarize the type of assessment/measurement according to the assessment period including number of times of measurement.

***Authors’ response***

***Changes made as suggested***

Statistical analyses
Page 9, for the statement 'Pearson correlation coefficients will be used to assess the ‘association’ between continuous exposure variables’ to be revised as to assess the ‘strength of association’.

Page 9 Paragraph 1, typo 4.7kP

***Authors’ response***

***Changes made as suggested***

Page 10, the description on the exploratory analyses of 'Vascular-parenchymal relationship in pAKI', 'Radiological-biomarker relationship in pAKI', 'Radiological-biomarker-biochemical relationship in pAKI' and statistical analyses' not clear and requires revision. What 3 times points refers to be clearly stated (i.e. pre and post or at each time point)

***Authors’ response***

***We apologize for the lack of clarity. We have rewritten the exploratory analyses referred to and clarified the 3 time points. (P:11, L: 1-15)***

Page 10, the write-up can be further improved by avoiding the use of ‘we plan’ too many times and could be written in passive form.

Page 9 Paragraph 1, typo 4.7kP

***Authors’ response***

***Changes made as suggested***

Data handling and security
Page 11, the sentence ‘Oversight of this investigation will be provided’ to be revised.

Figure 1 requires improvement. The figure is difficult to be visualized.

References to conform with the journal format.

***Authors’ response***

***We have changed the statement as suggested (P:12, L:21-23)***

***We have also modified the figure and made the change in the legend***

7. PLOS authors have the option to publish the peer review history of their article ([what does this mean?](https://journals.plos.org/plosone/s/editorial-and-peer-review-process#loc-peer-review-history)). If published, this will include your full peer review and any attached files.


If you choose “no”, your identity will remain anonymous but your review may still be made public.


**Do you want your identity to be public for this peer review?** For information about this choice, including consent withdrawal, please see our [Privacy Policy](https://www.plos.org/privacy-policy).

Reviewer #1: No

Reviewer #2: No

Reviewer #3: No
