# Supplementary material for: The bio-sonographic index. A novel modality for early detection of acute kidney injury after complex vascular surgery. A protocol for an exploratory prospective study
Source: PLoS One. 2020 Nov 17;15(11):e0241782. doi: 10.1371/journal.pone.0241782 (PMC7671487; doi:10.1371/journal.pone.0241782)
Supplement: Attachment — Submitted filename: response to the reviewer_ DB edit _AT.docx [file pone.0241782.s002.docx]

Re: PONE-D-20-08584
The Bio-sonographic Index. A Novel Modality for Early Detection of Acute Kidney Injury after Complex Vascular Surgery. A Protocol for an Exploratory Prospective Study
PLOS ONE

Dear Dr. Hodge,

We would like to thank the reviewers for their insightful comments on our manuscript.

We have revised the manuscript to address all the reviewers’ comments. We have added 2 new references to support our additions.

Please see attached are:

1. Revised manuscript in track changes
2. Revised manuscript not in track changes
3. Rebuttal letter addressing reviewers’ comments one by one

We hope that you now find our manuscript now suitable for publication at PLOS one.

Sincerely,

Ahmed Zaky

**Comments to the Author**

1. Does the manuscript provide a valid rationale for the proposed study, with clearly identified and justified research questions?

The research question outlined is expected to address a valid academic problem or topic and contribute to the base of knowledge in the field.

Reviewer #1: Yes

Reviewer #2: Yes

2. Is the protocol technically sound and planned in a manner that will lead to a meaningful outcome and allow testing the stated hypotheses?

The manuscript should describe the methods in sufficient detail to prevent undisclosed flexibility in the experimental procedure or analysis pipeline, including sufficient outcome-neutral conditions (e.g. necessary controls, absence of floor or ceiling effects) to test the proposed hypotheses and a statistical power analysis where applicable. As there may be aspects of the methodology and analysis which can only be refined once the work is undertaken, authors should outline potential assumptions and explicitly describe what aspects of the proposed analyses, if any, are exploratory.

Reviewer #1: Yes

Reviewer #2: Yes

3. Is the methodology feasible and described in sufficient detail to allow the work to be replicable?

Reviewer #1: Yes

Reviewer #2: Yes

4. Have the authors described where all data underlying the findings will be made available when the study is complete?

The [PLOS Data policy](https://urldefense.proofpoint.com/v2/url?u=https-3A__journals.plos.org_plosone_s_materials-2Dand-2Dsoftware-2Dsharing&d=DwMGaQ&c=o3PTkfaYAd6-No7SurnLt5qpge1aKYwPQyBFS7c8AA0&r=TeljxBtNrT-s2ZLhZ6Crcw&m=ToRHgOXsss6LvSV222bUONSnG7OvM1WJWhk2PfXTigs&s=MzsEHsSxTLYMMOBdBdTurnfrt8SsFV7_kfwZHNfvX0o&e=) requires authors to make all data underlying the findings described in their manuscript fully available without restriction, with rare exception, at the time of publication. The data should be provided as part of the manuscript or its supporting information, or deposited to a public repository. For example, in addition to summary statistics, the data points behind means, medians and variance measures should be available. If there are restrictions on publicly sharing data—e.g. participant privacy or use of data from a third party—those must be specified.

Reviewer #1: Yes

Reviewer #2: Yes

5. Is the manuscript presented in an intelligible fashion and written in standard English?

*PLOS ONE* does not copyedit accepted manuscripts, so the language in submitted articles must be clear, correct, and unambiguous. Any typographical or grammatical errors should be corrected at revision, so please note any specific errors here.

Reviewer #1: Yes

Reviewer #2: Yes

6. Review Comments to the Author

Please use the space provided to explain your answers to the questions above and, if applicable, provide comments about issues authors must address before this protocol can be accepted for publication. You may also include additional comments for the author, including concerns about research or publication ethics.

You may also provide optional suggestions and comments to authors that they might find helpful in planning their study.


(Please upload your review as an attachment if it exceeds 20,000 characters)

Reviewer #1: the manuscript provide a valid rationale for the proposed study, with clearly identified and justified research questions. I have no additional comments about this manuscript

***Authors’ response***

***We thank the reviewer for his comment***

Reviewer #2: Thank you for allowing me to review the changes made by Dr. Zaky et. al in their protocol. I commend the authors on their revisions, and I think this version is an improvement. I continue to think the overall aims of the project are valuable, and I have only a few comments that I hope will help with the manuscript.

BSI Components
1. I appreciate the further clarification of the BSI components (10% increase from pre-surgical values in either SWE, Nephrocheck, or NGAL). However, there is no data given to support these cut-points, and it appears to me that this study is expected to generate some of that data. If it is indeed the case that the cutpoints might change based on your findings, it might be useful to explain how you intend to approach creation of the BSI score in the methods, rather than giving specific cut points a priori.

***Authors’ response***

**We thank the reviewer for his comment.** ***We chose the 10% increase rather than the 0.3 cut off values for cell cycle arrest biomarkers to be consistent with the lack of cut off values for the other components of the BSI. Furthermore, the cut off values of the cell cycle arrest biomarkers are unknown in the preoperative period. Additionally, there is recent evidence to suggest a prediction of AKI at cut off values of less than 0.3 (>0.8 <0.2). We added a paragraph to reflect this justification in the discussion (Pages 17, 18, lines 369-379). We have also alluded to the exploratory nature of this index in the discussion.***

2. I know UAB is a leader in NGAL use, but I would be concerned about using that test in this population. NGAL is well known to increase in high-inflammatory states due to production from extra-renal sources. NGAL is likely to go up simply from post-surgical inflammation. I see this was added based on Reviewer #1’s recommendation, but I suspect it will not be a useful addition, personally. I suppose given that this study is designed to optimize the BSI, you could see whether NGAL adds value to the BSI or not.

***Authors’ response***

***We thank the reviewer for his comment. We included NGAL to explore its role in this population and its trend among open and endovascularly approached aneurysm repairs. as such, the BSI, will be amenable to further refinements in the biomarkers as it is validated in larger group of patients.***

3. Nephrocheck has a fairly standard value of 0.3 (as in the PREV-AKI trial, for instance). The 10% change is a metric I’ve not seen before with that marker.

***Authors’ response***

***We chose the 10% increase rather than the 0.3 cut off values for cell cycle arrest biomarkers to be consistent with the lack of cut off values for the other components of the BSI. Furthermore, the cut off values of the cell cycle arrest biomarkers are unknown in the preoperative period. additionally, there is recent evidence to suggest a prediction of AKI at cut of values of less than 0.3 (>0.8 <0.2). We have added a paragraph to reflect this justification in the discussion (Pages 17, 18, lines 369-379).***

4. I continue to be skeptical that there will be significant SWE changes that occur as early as 6 to 24 hours, but I suppose we will find out. I appreciate the inclusion of additional references and discussion.

Sample Size Calculation

5. 40% strikes me as a very high estimate of post-operative AKI, especially given the study design, and doesn’t seem supported by the references provided.
a. Reference 1, Hobson et al.: EVAR: 5.5% – 18%; Branched or fenestrated AAA: 28%; TEVAR: 9.7% (30% after branched aortic dissections); Huber et al. (cited within reference 1) had an AKI rate of 49%, but this seems to be driven by a high number of emergency cases. Emergency cases are an exclusion criteria in this study.
b. Reference 2, Martin-Gonzalez et al. Fenestrated and Branched Endografting: 29% AKI
c. Reference 3, Lee et al; Snorkel approach for EVAR: 32.6% AKI
d. Reference 4, Saratiz et al. Elective EVAR: 18.8% AKI
e. We recently published a study showing a 20.8% AKI rate following elective or urgent aneurysm repair, and several other studies I pulled up in a brief lit search showed AKI rates closer to 15-25% depending on the exact procedure (in elective cases).

My overall concern is that this study will be underpowered with only 80 patients.

***Authors’ response***

**We thank the reviewer for his comment and agree with his concerns. We based our sample size calculation on several factors. First, our population spans open thoraco-abdominal and abdominal aneurysms repairs known to have a higher incidence of pAKI compared with the endovascular approaches. We have added a new reference (reference 19) that shows the relatively high incidence of pAKI in this population. Second, we have conducted a retrospective analysis of the incidence of pAKI after complex open and endovascular aneurysm repairs at our institution and found an incidence suggestive of 40%. We have made changes in the manuscript (p 6, L 121- L123) to justify our viewpoint.**

6. The language regarding “expected to develop AKI” is confusing. I assume that this is referring to patients who have a positive BSI compared to those who have a negative BSI? I think it would be more clear to say that you expect that 70% of patients with a positive BSI will go on to develop AKI.

***Authors’ response:***

***We apologize for the confusion. The change is now made as suggested (P6, L 1211-126).***

7. PLOS authors have the option to publish the peer review history of their article ([what does this mean?](https://urldefense.proofpoint.com/v2/url?u=https-3A__journals.plos.org_plosone_s_editorial-2Dand-2Dpeer-2Dreview-2Dprocess-23loc-2Dpeer-2Dreview-2Dhistory&d=DwMGaQ&c=o3PTkfaYAd6-No7SurnLt5qpge1aKYwPQyBFS7c8AA0&r=TeljxBtNrT-s2ZLhZ6Crcw&m=ToRHgOXsss6LvSV222bUONSnG7OvM1WJWhk2PfXTigs&s=H_QYZpvn3UV4IIi8Jpxt2QEe24uJJzNortjQQRjIGmI&e=)). If published, this will include your full peer review and any attached files.


If you choose “no”, your identity will remain anonymous but your review may still be made public.


**Do you want your identity to be public for this peer review?** For information about this choice, including consent withdrawal, please see our [Privacy Policy](https://urldefense.proofpoint.com/v2/url?u=https-3A__www.plos.org_privacy-2Dpolicy&d=DwMGaQ&c=o3PTkfaYAd6-No7SurnLt5qpge1aKYwPQyBFS7c8AA0&r=TeljxBtNrT-s2ZLhZ6Crcw&m=ToRHgOXsss6LvSV222bUONSnG7OvM1WJWhk2PfXTigs&s=z2rLavtDM2gcuW6UYUFZdPHIXjUgirr5Ar5s8zzEwO4&e=).

Reviewer #1: No

Reviewer #2: No
